# Comparison of Thoracic Segmental Spinal Anesthesia Using Isobaric Levobupivacaine With Fentanyl Versus Dexmedetomidine in Patients Undergoing Laparoscopic Cholecystectomy

**DOI:** 10.7759/cureus.109732

**Published:** 2026-05-27

**Authors:** Samrat Arya, Bhawna Singh, Mohd Khalik

**Affiliations:** 1 Department of Anesthesiology, Rohilkhand Medical College and Hospital, Bareilly, IND

**Keywords:** dexmedetomidine, fentanyl, laparoscopic cholecystectomy, levobupivacaine, thoracic segmental spinal anesthesia

## Abstract

Introduction: Thoracic segmental spinal anesthesia is increasingly used as an alternative to general anesthesia for laparoscopic cholecystectomy because of its potential benefits in providing effective intraoperative anesthesia and improved postoperative recovery. The present study aimed to compare the hemodynamic effects, block characteristics, and postoperative analgesia effects of isobaric levobupivacaine combined with fentanyl versus dexmedetomidine in thoracic segmental spinal anesthesia.

Materials and methods: In this prospective comparative study, 60 patients who underwent elective laparoscopic cholecystectomy were divided into two groups of 30 each. Levobupivacaine was administered intrathecally with fentanyl (25 µg) in one group and levobupivacaine and dexmedetomidine (5 µg) in the other. Hemodynamic parameters, duration of analgesia, features of sensory and motor block, and side effects were recorded. The Chi-square/Fisher's exact test and the independent samples t-test were used for statistical analysis. A statistically significant p-value is one that is less than 0.05.

Results: Baseline characteristics were comparable between the groups. The onset of sensory block was significantly faster in the fentanyl group (3.46 ± 1.09 min) compared to the dexmedetomidine group (3.96 ± 0.77 min, p = 0.045). However, the duration of sensory block (190.43 ± 8.48 vs. 157.76 ± 16.31 min, p < 0.001) and duration of analgesia (279.43 ± 30.49 vs. 183.03 ± 20.87 min, p < 0.001) were significantly longer in the dexmedetomidine group. Hemodynamic parameters remained stable in both groups, although dexmedetomidine was associated with greater reductions in heart rate and blood pressure during the early intraoperative period. The incidence of adverse effects was comparable, with no cases of postoperative nausea and vomiting observed.

Conclusion: Dexmedetomidine demonstrated superior efficacy as an intrathecal adjuvant by significantly prolonging sensory blockade and postoperative analgesia compared to fentanyl, albeit with a modest increase in manageable hemodynamic effects. These findings support its preferential use in laparoscopic cholecystectomy, where prolonged pain relief and stable intraoperative conditions are desired.

## Introduction

Thoracic segmental spinal anesthesia (TSSA) has gained increasing attention as a viable alternative to general anesthesia for selected upper abdominal surgeries, particularly laparoscopic cholecystectomy [1]. With the growing emphasis on minimally invasive procedures, enhanced recovery protocols, and reduced perioperative morbidity, regional anesthesia techniques that provide effective analgesia with minimal systemic effects are being actively explored. TSSA involves targeted administration of local anesthetic agents into the thoracic subarachnoid space, producing a segmental block that corresponds to the dermatomes involved in surgery [1,2]. This approach offers several advantages, including avoidance of airway manipulation, preservation of spontaneous respiration, reduced postoperative nausea and vomiting, and improved postoperative analgesia.

Levobupivacaine has emerged as the preferred local anesthetic because of its comparable efficacy and improved safety profile, particularly with reduced cardiotoxicity and neurotoxicity. When used in its isobaric form, it provides a predictable spread within the cerebrospinal fluid, allowing better control over the extent of the blockade. However, the duration of analgesia with local anesthetic alone may be limited, necessitating the use of intrathecal adjuvants to enhance the block characteristics [3,4].

Fentanyl, a lipophilic opioid, is widely used as an adjuvant because of its rapid onset and ability to enhance sensory blockade without significantly prolonging motor block [5]. In contrast, dexmedetomidine, a highly selective α2-adrenergic agonist, prolongs both sensory and motor blockade and provides extended postoperative analgesia through its central and peripheral actions [6]. While fentanyl offers a faster onset and hemodynamic stability, dexmedetomidine provides a longer duration of analgesia but may be associated with bradycardia and hypotension [6]. Given these differences, comparing these two adjuvants in thoracic spinal anesthesia is clinically relevant for determining the optimal agent that balances rapid onset, adequate duration, hemodynamic stability, and patient comfort in laparoscopic cholecystectomy.

In patients having laparoscopic cholecystectomy, this study compared the hemodynamic effects, intraoperative stability, and postoperative analgesia of thoracic segmental spinal anesthesia utilizing isobaric levobupivacaine with fentanyl against isobaric levobupivacaine with dexmedetomidine. The primary objective of the present study was to compare the duration of postoperative analgesia between intrathecal dexmedetomidine and fentanyl used as adjuvants to isobaric levobupivacaine in thoracic segmental spinal anesthesia for laparoscopic cholecystectomy. Secondary objectives included comparison of hemodynamic parameters, sensory and motor block characteristics, postoperative pain scores, and adverse effects between the two groups. We hypothesized that dexmedetomidine would prolong postoperative analgesia and sensory blockade compared with fentanyl without causing clinically significant hemodynamic instability. This study was conducted to help inform the choice of intrathecal adjuvant in patients undergoing laparoscopic cholecystectomy under thoracic segmental spinal anesthesia.

## Materials and methods

This prospective comparative study was conducted in the Department of Anesthesiology at Rohilkhand Medical College and Hospital, Bareilly, Uttar Pradesh, India, over a period of one year from April 2024 to April 2025. The study was initiated after obtaining approval from the Institutional Ethics Committee of the Rohilkhand Medical College and Hospital (Approval No.: IEC/RMCH/13/2024/APR dated 1/04/24). Written informed consent was obtained from all participants prior to enrollment. The study was designed as a comparative clinical evaluation rather than a randomized controlled trial, as patient allocation into groups was performed in a pragmatic, non-randomized manner based on clinical workflow and drug availability, thereby reflecting routine anesthesia practice. Patients were allocated pragmatically based on anesthesiologist preference and availability of the study drugs at the time of surgery.

The study comprised 60 adult patients scheduled for elective laparoscopic cholecystectomy, ages 18 to 65. If a patient was judged clinically fit to have laparoscopic surgery and spinal anesthesia, they were eligible. Pregnancy, infection at the intended spinal injection site, coagulation problems, severe hypovolemia, elevated intracranial pressure, and a known allergy or hypersensitivity to levobupivacaine, fentanyl, or dexmedetomidine were all grounds for exclusion.

The sample size was calculated using a priori power analysis with the G*Power software (version 3.1, Heinrich Heine University Düsseldorf, Düsseldorf, Germany), considering the duration of analgesia as the primary outcome variable. With an effect size of 0.56 from a previous study for the duration of the anesthesia variable, a power of 80%, and an alpha error of 5%, the minimum sample size required was 27 patients per group [7]. To account for possible dropouts and improve statistical validity, 30 patients were included in each group, resulting in a total sample size of 60 participants.

All patients underwent a detailed preanesthetic evaluation, including history, systemic examination, and airway assessment. The baseline hemodynamic parameters were recorded. Patients were kept NPO (nil per os) for at least 8 hours prior to surgery. Premedication included alprazolam (Alprax®; Torrent Pharmaceuticals Ltd., Ahmedabad, India) and ranitidine (Rantac®; J.B. Chemicals & Pharmaceuticals Ltd., Mumbai, India).

In the operating theater, standard monitoring (ECG, NIBP, SpO₂) was instituted. An 18-G intravenous cannula was secured, and Ringer's lactate (RL Infusion®, Baxter India Pvt. Ltd., Gurgaon, India) was administered. Under strict aseptic precautions, thoracic segmental spinal anesthesia was performed in the sitting position using a 25-G Quincke spinal needle at the T10-T11 or T11-T12 interspace. After confirming free cerebrospinal fluid flow, isobaric levobupivacaine 0.5% (Levo-Anawin®, Neon Laboratories Ltd., Mumbai, India) 10 mg (2 ml) was administered intrathecally along with either fentanyl citrate (Fentanyl Citrate Injection®, Troikaa Pharmaceuticals Ltd., Ahmedabad, India) 25 µg (0.5 ml) or dexmedetomidine (Dexem®, Themis Medicare Ltd., Mumbai, India) 5 µg diluted to 0.5 ml, depending on the group. Patients were then placed in the supine position, and oxygen supplementation was provided at 4-6 L/min.

Sensory blockade was assessed using the pinprick method at 2-minute intervals until the desired dermatome level was achieved. Motor blockade was assessed by observing the degree of motor impairment in the lower extremities and categorized according to the Bromage motor block grading system. Hemodynamic parameters, including heart rate, systolic blood pressure, diastolic blood pressure, mean arterial pressure, and oxygen saturation, were recorded at predefined intraoperative intervals. The onset and duration of sensory and motor blockade, time to maximum block height, and duration of postoperative analgesia were documented. Postoperative pain assessment was based on patient-reported pain perception and the time to first requirement of rescue analgesia. Adverse events, including hypotension, bradycardia, nausea, vomiting, pruritus, and shivering, were monitored throughout the intraoperative and early postoperative period.

Microsoft Excel (Microsoft Corporation, Redmond, WA, USA) was used to enter the data, and IBM Corp. Released 2019. IBM SPSS Statistics for Windows, Version 25. Armonk, NY: IBM Corp. was used for analysis. The mean ± standard deviation is used to represent continuous variables, whereas frequencies and percentages are used to portray categorical variables. Using the Shapiro-Wilk test, normality was evaluated. Repeated-measures analysis of variance (ANOVA) was employed for hemodynamic variables, and independent samples t-test and Chi-square/Fisher's exact test were utilized when needed. P <0.05 was used to determine statistical significance.

## Results

A total of 60 patients were included in the study, with equal distribution between the two groups. Baseline demographic characteristics were comparable between the groups, with no statistically significant differences, ensuring the homogeneity of the study population (Table 1).

**Table 1:** Demographic characteristics of study participants. BMI: Body mass index, p > 0.05 denotes no statistical significance. Continuous data are presented as mean and standard deviation, whereas categorical data are presented as frequency (n) percentage (%). An independent sample t-test (t) was applied for continuous variables, and a chi-square test (χ²) was applied for categorical variables.

Parameter	Fentanyl group (n = 30)	Dexmedetomidine group (n = 30)	Test value	p-value
Age (years), Mean +SD	46.87 ± 10.70	43.73 ± 12.36	t = 1.05	0.297
Male, n (%)	6 (20.00)	8 (26.67)	χ² = 0.37	0.540
Female, n (%)	24 (80.00)	22 (73.33)		
BMI (kg/m²), mean ± SD	25.13 ± 3.92	25.20 ± 3.52	t = 0.07	0.941
Height (cm), mean ± SD	161.80 ± 8.18	163.43 ± 7.27	t = 0.82	0.410
Weight (kg), mean ± SD	65.07 ± 7.86	67.27 ± 8.39	t = 1.05	0.292

Regarding anesthetic characteristics, a statistically significant difference was observed in the onset and duration of sensory blockade as well as the duration of analgesia. The fentanyl group demonstrated a faster onset of sensory block, whereas the dexmedetomidine group showed a significantly prolonged duration of sensory blockade and postoperative analgesia. The time to achieve maximum block height, distribution of ASA grades, interspace levels, and maximum dermatome level was comparable between the two groups (Table 2).

**Table 2:** Comparison of anesthetic characteristics between groups. Data are presented as mean ± standard deviation or number (percentage), ASA: American Society of Anesthesiologists, *Statistically significant (p < 0.05). An independent sample t-test (t) was applied for continuous variables and a chi-square test (χ²) was applied for categorical variables.

Parameter	Group		Test value	p-value
	Fentanyl	Dexmedetomidine		
Duration of analgesia (min), mean ± SD	183.03 ± 20.87	279.43 ± 30.49	t = 14.20	< 0.001*
Onset of sensory block (min), mean ± SD	3.46 ± 1.09	3.96 ± 0.77	t = 2.05	0.045*
Duration of sensory block (min), mean ± SD	157.76 ± 16.31	190.43 ± 8.48	t = 9.87	< 0.001*
Time to maximum block height (min), mean ± SD	8.07 ± 2.08	7.30 ± 1.12	t = 1.79	0.078
ASA grade I, n (%)	19 (63.33)	23 (76.67)	χ² = 1.41	0.231
ASA grade II, n (%)	11 (36.67)	7 (23.33)		
Interspace T10–T11, n (%)	10 (33.33)	12 (40.00)	χ² = 0.29	0.591
Interspace T11–T12, n (%)	20 (66.67)	18 (60.00)		
Maximum dermatome level T3, n (%)	5 (16.67)	5 (16.67)	χ² = 0.00	0.997
Maximum dermatome level T4, n (%)	25 (83.33)	25 (83.33)		

Motor blockade characteristics revealed a significant difference in the intensity of the motor block following spinal administration, with dexmedetomidine producing a more profound initial motor blockade. However, motor recovery at the end of surgery was comparable between groups. The incidences of hypotension and bradycardia were higher in the dexmedetomidine group, although this difference was not statistically significant. No cases of shivering, pruritus, or postoperative nausea or vomiting were observed in either group (Table 3).

**Table 3:** Comparison of clinical complications between groups. Data are presented as numbers (percentages), *statistically significant (p < 0.05), NA: not applicable, and the Chi-square test or Fisher’s exact test is used as appropriate.

Parameter	Category	Group		Chi or Fisher test	p-value
		Fentanyl	Dexmedetomidine		
Motor block score (Bromage after spinal)	1	5 (16.67)	2 (6.67)	6.25	0.045*
	2	17 (56.67)	9 (30.00)		
	3	8 (26.67)	19 (63.33)		
Motor function at the end of surgery	0	17 (56.67)	12 (40.00)	1.44	0.230
	1	13 (43.33)	18 (60.00)		
Hypotension	No	28 (93.33)	23 (76.67)	3.27	0.070
	Yes	2 (6.67)	7 (23.33)		
Bradycardia	No	28 (93.33)	23 (76.67)	3.27	0.070
	Yes	2 (6.67)	7 (23.33)		
Shivering	No	30 (100)	30 (100)	-	NA
	Yes	0 (0)	0 (0)		
Pruritus	No	30 (100)	30 (100)	-	NA
	Yes	0 (0)	0 (0)		
Postoperative nausea and vomiting	No	30 (100)	30 (100)	-	NA
	Yes	0 (0)	0 (0)		

**Figure 1:**
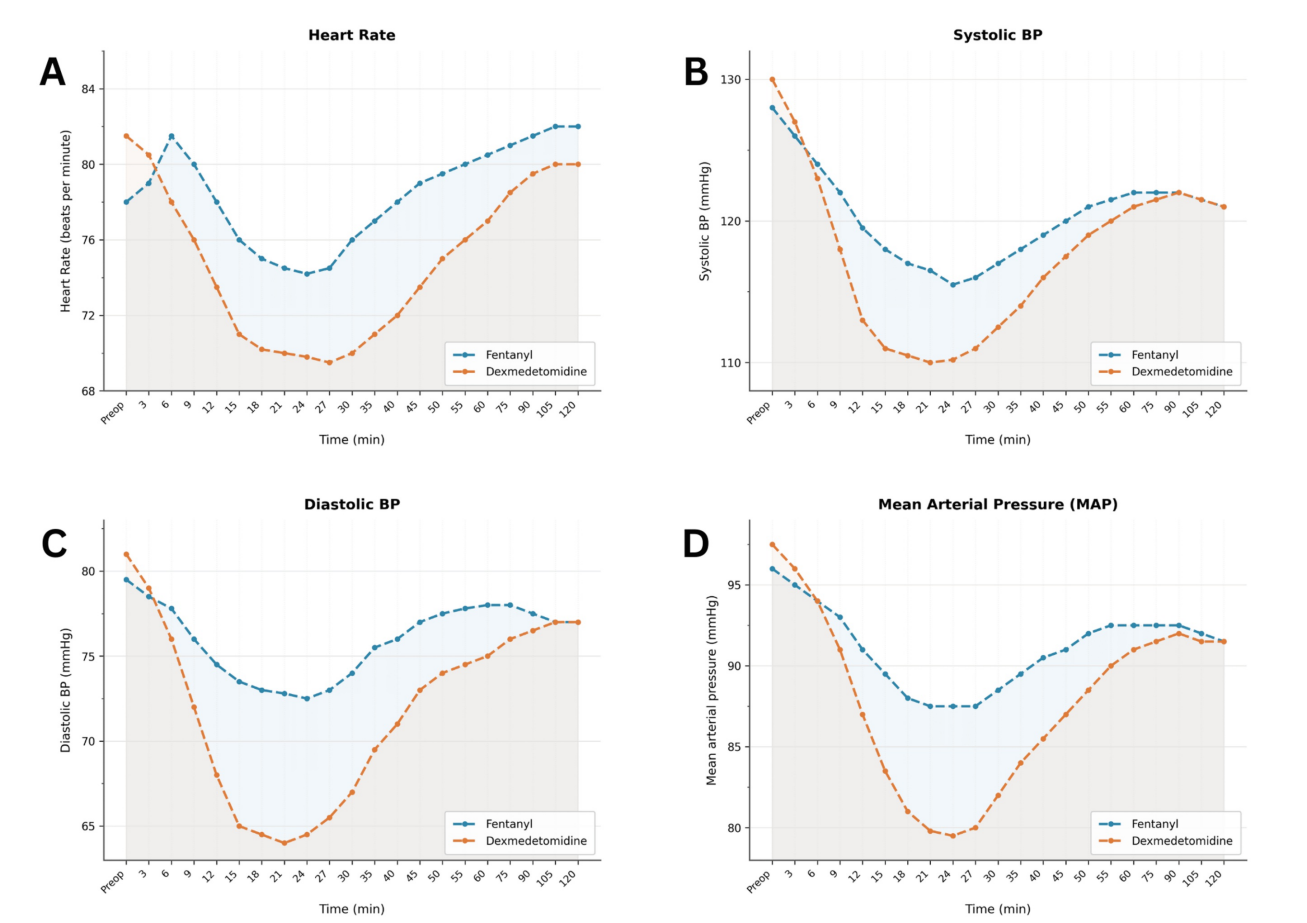
Hemodynamic parameters over time in both groups: (A) heart rate; (B) systolic BP; (C) diastolic BP; (D) mean arterial pressure. Graphical representation of intraoperative hemodynamic parameters showing changes in heart rate, systolic blood pressure (BP), diastolic blood pressure, and mean arterial pressure (MAP) in patients receiving isobaric levobupivacaine with fentanyl and dexmedetomidine.

Analysis of hemodynamic parameters demonstrated similar baseline values in both groups. Following spinal anesthesia, the dexmedetomidine group exhibited a greater reduction in heart rate and blood pressure during the early intraoperative period than the fentanyl group. However, all parameters remained within the clinically acceptable limits and stabilized overtime in both groups, with no requirement for major intervention (Figure 1). Overall, dexmedetomidine provided prolonged analgesia and enhanced sensory blockade, whereas fentanyl was associated with a faster onset and relatively stable hemodynamic profile.

## Discussion

The present study evaluated the comparative efficacy of fentanyl and dexmedetomidine as intrathecal adjuvants to isobaric levobupivacaine in thoracic segmental spinal anesthesia for laparoscopic cholecystectomy. These findings demonstrate that while fentanyl provides a faster onset of sensory blockade, dexmedetomidine significantly prolongs the duration of sensory block and postoperative analgesia, with acceptable hemodynamic stability.

The faster onset of the sensory blockade observed with fentanyl can be attributed to its high lipophilicity, which facilitates rapid diffusion across neural tissues and early binding to μ-opioid receptors in the dorsal horn. This results in the prompt modulation of nociceptive transmission and quicker establishment of anesthesia. Similar findings have been reported by Dahlgren et al. [8], who demonstrated that intrathecal fentanyl enhances the quality and onset of spinal anesthesia when added to bupivacaine. Likewise, Palmer et al. [9] reported that subarachnoid fentanyl significantly augmented the efficacy of spinal anesthesia by improving intraoperative analgesia and reducing the requirement for supplemental anesthetic agents. These findings support the role of fentanyl as an effective adjuvant for achieving rapid and reliable block characteristics.

Similar findings were reported by Ben-David et al. [10], who demonstrated that intrathecal fentanyl accelerates the onset of spinal anesthesia when combined with local anesthetics. Additionally, Hunt et al. [11] reported enhanced onset characteristics with fentanyl due to its rapid spinal cord penetration and receptor affinity. In contrast, dexmedetomidine significantly prolonged the duration of the sensory blockade and analgesia. This effect can be explained by its action as a highly selective α2-adrenergic agonist that produces both presynaptic inhibition of neurotransmitter release and postsynaptic hyperpolarization of dorsal horn neurons. This dual mechanism enhances and prolongs the effect of local anesthetics [6]. Gupta et al. [12] reported similar findings, demonstrating that dexmedetomidine significantly prolongs sensory and motor block durations compared to fentanyl. Similarly, Al-Mustafa et al. [13] observed a dose-dependent prolongation of spinal anesthesia with dexmedetomidine, reinforcing its role as an effective intrathecal adjuvant.

The prolonged duration of analgesia in the dexmedetomidine group observed in this study has important clinical implications. Prolonged postoperative analgesia reduces the need for rescue analgesics, improves patient comfort, and enhances recovery profiles, particularly in minimally invasive procedures, such as laparoscopic cholecystectomy. Mahendru et al. [14] observed that intrathecal dexmedetomidine significantly prolonged both sensory and motor blockade compared with fentanyl and clonidine. Rahimzadeh et al. [15] also demonstrated superior analgesic duration and improved block characteristics with dexmedetomidine compared with fentanyl in lower limb surgeries. Furthermore, Kalbande et al. [16] reported that dexmedetomidine provided prolonged postoperative analgesia and improved block quality with acceptable hemodynamic stability, reinforcing its role as a superior intrathecal adjuvant. These findings are consistent with those of the present study and support the use of dexmedetomidine in procedures where prolonged analgesia is desirable.

The motor blockade characteristics revealed that dexmedetomidine produced a more profound initial motor block than fentanyl. This is consistent with the known pharmacological action of α2-agonists, which enhance local anesthetic effects on both sensory and motor fibers [14-16]. However, motor recovery at the end of surgery was comparable between the groups, suggesting that prolonged block did not significantly delay postoperative recovery. This balance between prolonged analgesia and acceptable motor recovery is advantageous in surgical settings, where early ambulation is desirable.

The hemodynamic parameters in both groups remained within clinically acceptable limits, although the dexmedetomidine group showed a greater reduction in heart rate and blood pressure during the early intraoperative period. This can be explained by its central sympatholytic effect, which leads to decreased norepinephrine release and reduced sympathetic tone. Despite this, no significant hemodynamic instability was observed, and the changes did not necessitate major interventions. Similar findings were reported by Khosravi et al. [17], who observed stable hemodynamics with dexmedetomidine despite mild bradycardia and hypotension. The ability of dexmedetomidine to attenuate stress response while maintaining stability is particularly beneficial in laparoscopic surgeries, where pneumoperitoneum can induce significant hemodynamic fluctuations.

The incidence of adverse effects in this study was low and comparable between groups. Although hypotension and bradycardia occurred more frequently in the dexmedetomidine group, the differences were not statistically significant. Importantly, no cases of postoperative nausea and vomiting, pruritus, or shivering were observed, indicating a favorable safety profile for both adjuvants. These findings are consistent with those of previous studies that reported minimal side effects with low-dose intrathecal dexmedetomidine and fentanyl [12,18].

Safari et al. [19] reported that intrathecal dexmedetomidine was associated with a lower incidence of postoperative nausea and vomiting than fentanyl when used as an adjuvant to bupivacaine. The reduced incidence of nausea and vomiting with dexmedetomidine may be attributed to its opioid-sparing effect and the lack of direct stimulation of chemoreceptor trigger zones, in contrast to opioid-based adjuvants. These properties make dexmedetomidine a favorable option in patients in whom minimizing postoperative nausea and vomiting is clinically desirable.

From a clinical perspective, the choice of adjuvant should be guided by surgical requirements and patient factors. Fentanyl may be preferred in situations requiring rapid onset and shorter duration, whereas dexmedetomidine is advantageous in procedures where prolonged analgesia and reduced postoperative analgesic requirements are desired. The enhanced analgesic profile and stable hemodynamics associated with dexmedetomidine make it a valuable adjuvant in thoracic spinal anesthesia for laparoscopic procedures.

The prolonged duration of postoperative analgesia observed with dexmedetomidine may have important clinical implications in laparoscopic cholecystectomy, where effective early postoperative pain control contributes to improved patient comfort and reduced analgesic requirement. Thoracic segmental spinal anesthesia offers additional potential advantages, including avoidance of airway manipulation, preservation of spontaneous ventilation, reduced postoperative nausea and vomiting, and attenuation of surgical stress responses. These benefits may be particularly relevant in selected patients where minimizing airway instrumentation or systemic opioid exposure is desirable. However, careful patient selection, vigilant monitoring, and familiarity with thoracic neuraxial techniques remain essential to ensure procedural safety.

However, certain limitations of the present study should be acknowledged. First, the study was conducted at a single center with a relatively modest sample size, which may limit the generalizability of the findings. Second, the pragmatic non-randomized and non-blinded study design may introduce selection bias, observer bias, and residual confounding despite comparable baseline characteristics between groups. Patient allocation was based on clinical workflow and drug availability, reflecting routine anesthesia practice but limiting the ability to establish definitive causal inferences. Third, the study was powered primarily for the duration of postoperative analgesia and may have been underpowered to detect clinically significant differences in adverse events and hemodynamic complications. In addition, postoperative sedation scores, patient satisfaction, rescue analgesic consumption, and long-term outcomes such as delayed neurological complications or urinary retention were not evaluated. Although thoracic segmental spinal anesthesia was performed safely in the present study, broader safety and feasibility considerations, including patient acceptance and rare neurological complications, require further evaluation in larger randomized multicenter studies with longer follow-up periods. Furthermore, subgroup analyses based on age, ASA status, or comorbidities were not performed due to the limited sample size. Therefore, the findings should be interpreted cautiously within the context of the observational comparative design.

## Conclusions

The present study demonstrated that intrathecal dexmedetomidine as an adjuvant to isobaric levobupivacaine in thoracic segmental spinal anesthesia was associated with prolonged sensory blockade and extended postoperative analgesia compared with fentanyl in patients undergoing laparoscopic cholecystectomy. In contrast, fentanyl provided a relatively faster onset of sensory blockade. Although dexmedetomidine was associated with greater reductions in heart rate and blood pressure during the early intraoperative period, these changes remained clinically manageable within the study setting. Both adjuvants demonstrated acceptable safety profiles without major adverse events or the requirement for conversion to general anesthesia. Within the limitations of this pragmatic comparative study, dexmedetomidine may offer potential advantages in procedures where prolonged postoperative analgesia is desirable. However, due to the non-randomized design, modest sample size, and single-center setting, the findings should be interpreted cautiously. Further large-scale randomized controlled studies with standardized postoperative assessment and longer follow-up are recommended to validate these findings and better establish the safety and clinical applicability of thoracic segmental spinal anesthesia with different intrathecal adjuvants.
